# Correction: Broadly directed SARS-CoV-2-specific CD4+ T cell response includes frequently detected peptide specificities within the membrane and nucleoprotein in patients with acute and resolved COVID-19

**DOI:** 10.1371/journal.ppat.1010220

**Published:** 2022-01-05

**Authors:** Janna Heide, Sophia Schulte, Matin Kohsar, Thomas Theo Brehm, Marissa Herrmann, Hendrik Karsten, Matthias Marget, Sven Peine, Alexandra M. Johansson, Alessandro Sette, Marc Lütgehetmann, William W. Kwok, John Sidney, Julian Schulze zur Wiesch

In Table 6, the 4th column of HLA alleles, the allele for DQB1 is missing a number. The correct column heading is: DQA1*05:01/DQB1*03:01. Please see the correct Table 6 below.

**Table 6:** In vitro binding capacity of 14 SARS-CoV-2-specific peptides to 22 frequent HLA class II MHC molecules. Binding capacities are expressed as IC50 nM values measured in classical *in vitro* binding assays based on inhibition of binding of a high affinity radiolabeled ligand to purified HLA molecules. High affinity binding is defined as IC50 < 1,000 nM and highlighted by bold font. For reasons of comprehensibility, values larger than 40,000 nM are indicated by a dash. The total number of alleles bound as well as the response frequency (RF) of the responding peptide is shown. Mem_P30 and Mem_P36, with the highest RFs, bound to 12 and 13 HLA molecules, respectively.

Peptide	aa position	Sequence															Alleles bound	RF	DPB1* 02:01	DPA1*01:03 DPB1*04:01	DQA1*05:01 DQB1*02:01	DQA1*05:01 DQB1*03:01	DQA1*03:01 DQB1*03:02	DQA1*01:01 DQB1*05:01	DQA1*01:02 DQB1*06:02
Env_11	46–60	L	V	K	P	S	F	Y	V	Y	S	R	V	K	N	L	13	36.1	**42**	**63**	-	5766	-	-	24387
Mem_30	146–160	R	G	H	L	R	I	A	G	H	H	L	G	R	C	D	12	72.2	**569**	**445**	-	15141	-	37659	-
Mem_34	166–180	K	E	I	T	V	A	T	S	R	T	L	S	Y	Y	K	13	41.7	1909	3337	13645	**649**	17391	-	1827
Mem_36	176–190	L	S	Y	Y	K	L	G	A	S	Q	R	V	A	G	D	13	66.7	13822	-	22448	**48**	-	15193	**428**
Ncl_11	51–65	S	W	F	T	A	L	T	Q	H	G	K	E	D	L	K	9	46.9	-	35760	30440	1304	19748	-	**952**
Ncl_17	81–95	D	D	Q	I	G	Y	Y	R	R	A	T	R	R	I	R	7	56.3	34655	-	-	5067	-	-	-
Ncl_18	86–100	Y	Y	R	R	A	T	R	R	I	R	G	G	D	G	K	6	56.3	-	-	-	6720	-	-	-
Ncl_22	106–120	P	R	W	Y	F	Y	Y	L	G	T	G	P	E	A	G	5	34.4	10053	-	**79**	1278	**414**	2239	7882
Ncl_25	121–135	L	P	Y	G	A	N	K	D	G	I	I	W	V	A	T	2	37.5	13013	20045	11706	14585	3795	-	**203**
Ncl_26	126–140	N	K	D	G	I	I	W	V	A	T	E	G	A	L	N	9	50	4140	8348	**422**	4027	**103**	-	2643
Ncl_44	216–230	D	A	A	L	A	L	L	L	L	D	R	L	N	Q	L	7	43.8	1162	2779	2487	22499	**860**	8458	2499
Ncl_45	221–235	L	L	L	L	D	R	L	N	Q	L	E	S	K	M	S	9	43.8	**285**	**701**	3315	-	11904	23089	8081
Ncl_54	266–280	K	A	Y	N	V	T	Q	A	F	G	R	R	G	P	E	9	43.8	8438	6056	-	**794**	33174	-	2936
Ncl_70	346–360	F	K	D	Q	V	I	L	L	N	K	H	I	D	A	Y	13	53.1	**287**	**794**	30656	33658	9176	6010	8602
