# Supplementary figures and images for: Broadly directed SARS-CoV-2-specific CD4+ T cell response includes frequently detected peptide specificities within the membrane and nucleoprotein in patients with acute and resolved COVID-19
Source: PLoS Pathog. 2021 Sep 16;17(9):e1009842. doi: 10.1371/journal.ppat.1009842 (PMC8445433; doi:10.1371/journal.ppat.1009842)

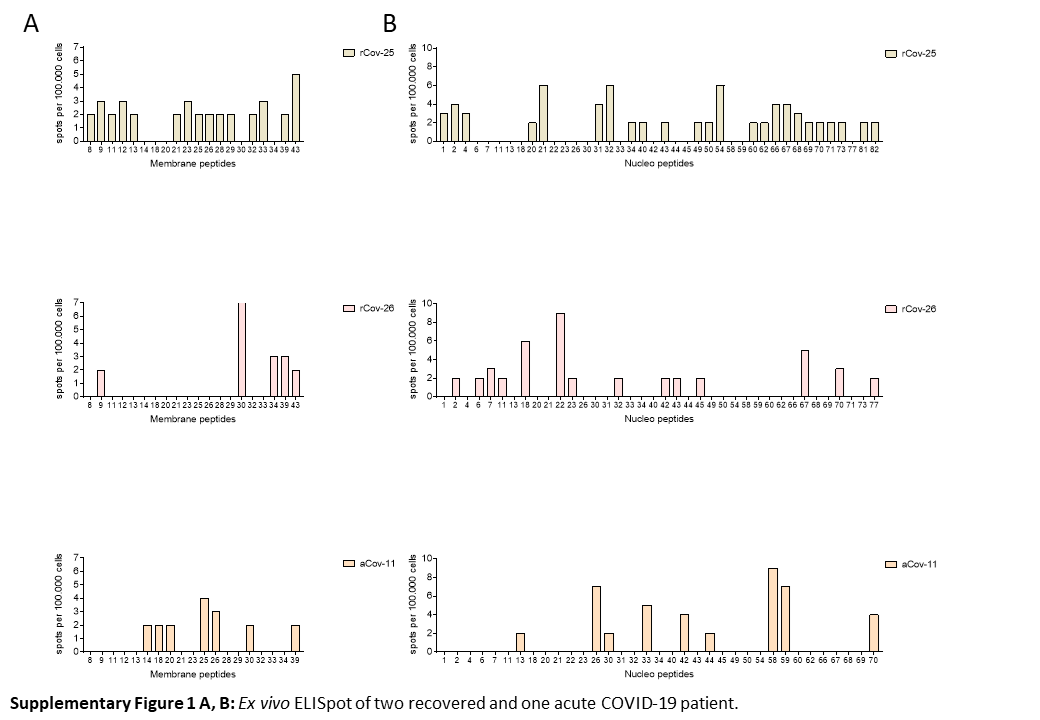

Supplement: S1 Fig — (A)Ex vivo ELISpot with PBMCs from two resolved and one acute COVID-19 patient stimulated with all 43 membrane peptides. The spots in the negative control ranged from 0 to 1 spot per 100.000 cells. (B) Ex vivo ELISpot with PBMCs from two resolved and one acute COVID-19 patients stimulated with all 82 nucleoprotein peptides. The spots in the negative control ranged from 0 to 1 spot per 100.000 cells. (TIF) [file ppat.1009842.s010.TIF]

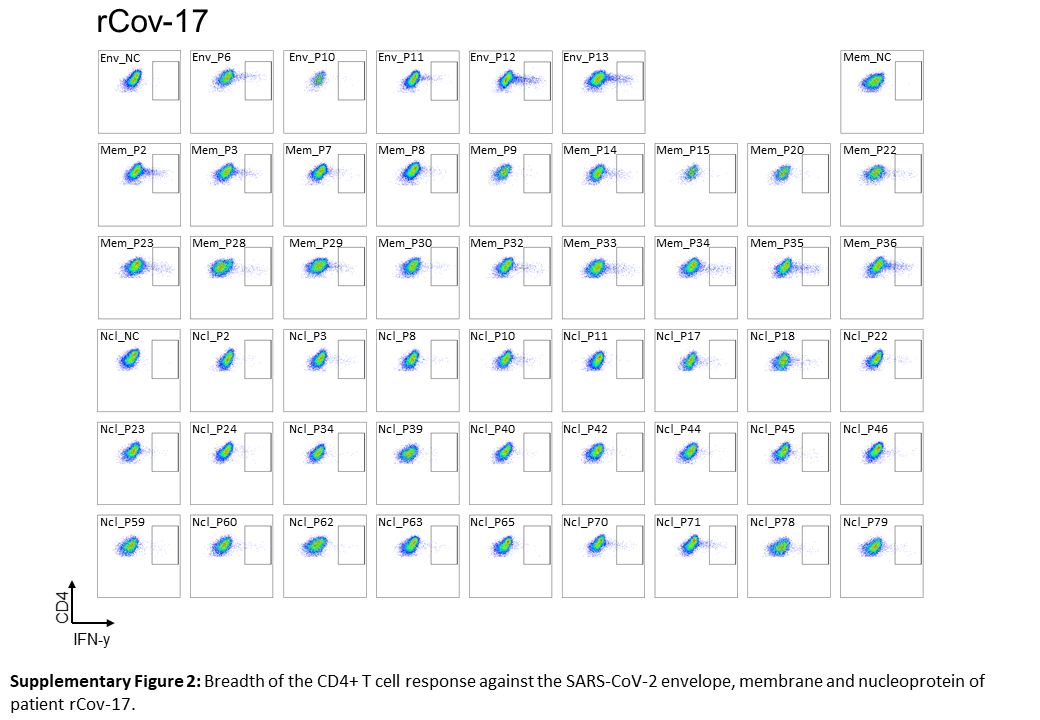

Supplement: S2 Fig — All IFN-y+ CD4+ T cell responses of patient rCov-17 against the envelope, membrane, and nucleoprotein. Gated on CD4+ T cells. All cytokine gates are set based on the respective negative control (R10 and DMSO). (TIF) [file ppat.1009842.s011.TIF]

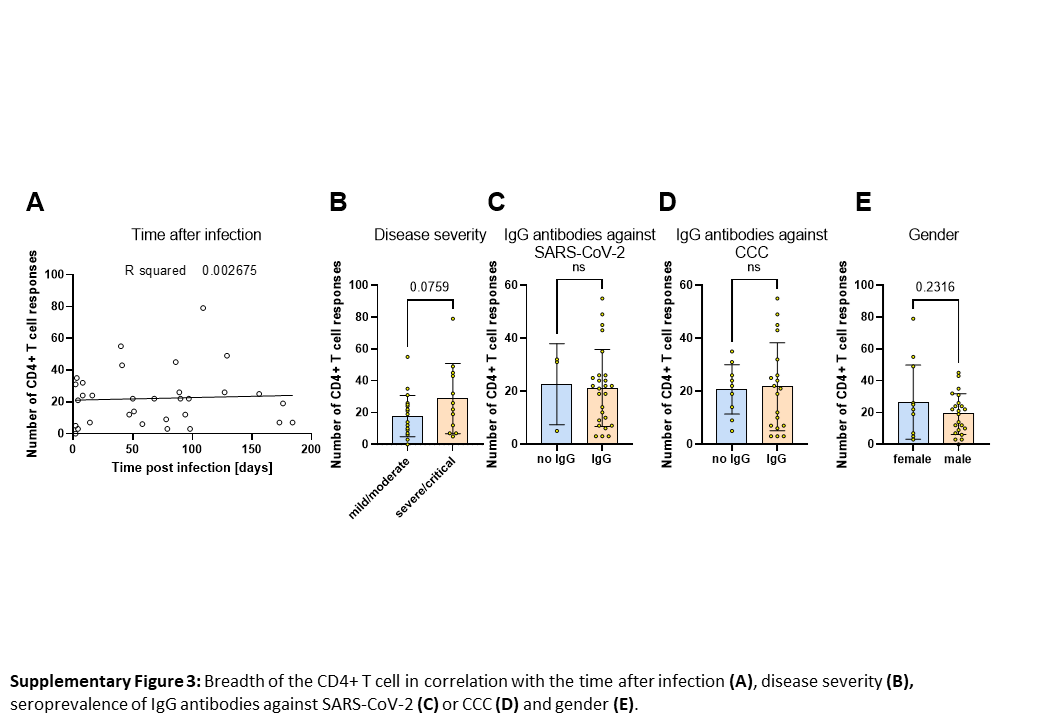

Supplement: S3 Fig — Breadth of the CD4+ T cell response in correlation with the time after infection (A), disease severity (B), seroprevalence of IgG antibodies against SARS-CoV-2 (C) or CCC (D) and gender (E). (TIF) [file ppat.1009842.s012.TIF]

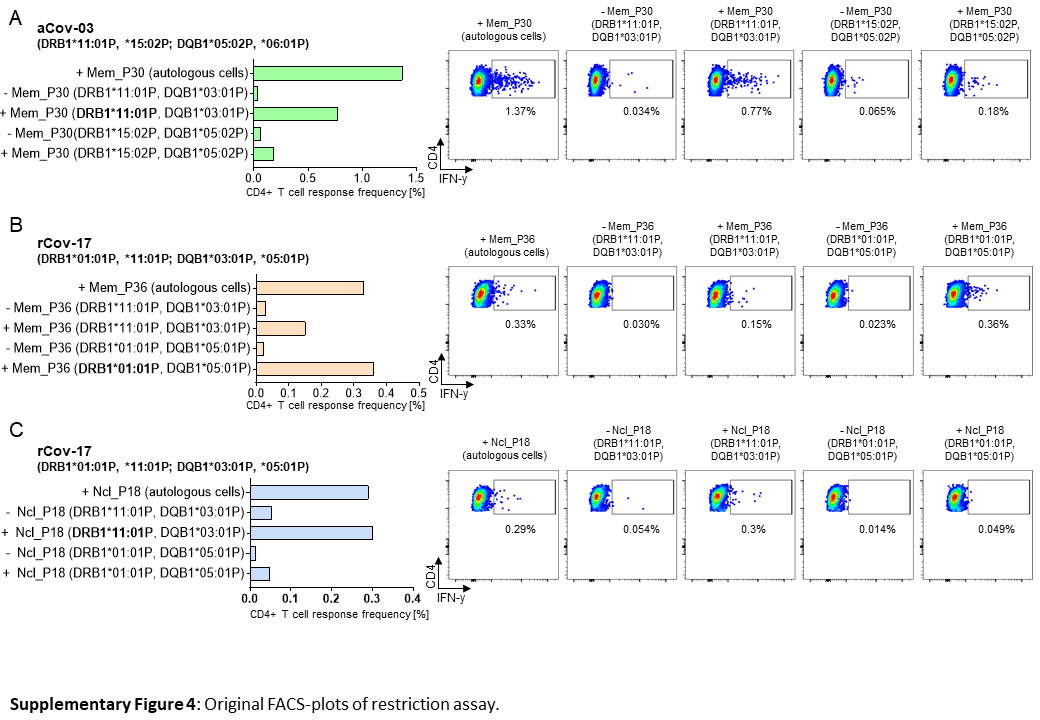

Supplement: S4 Fig — (TIF) [file ppat.1009842.s013.TIF]

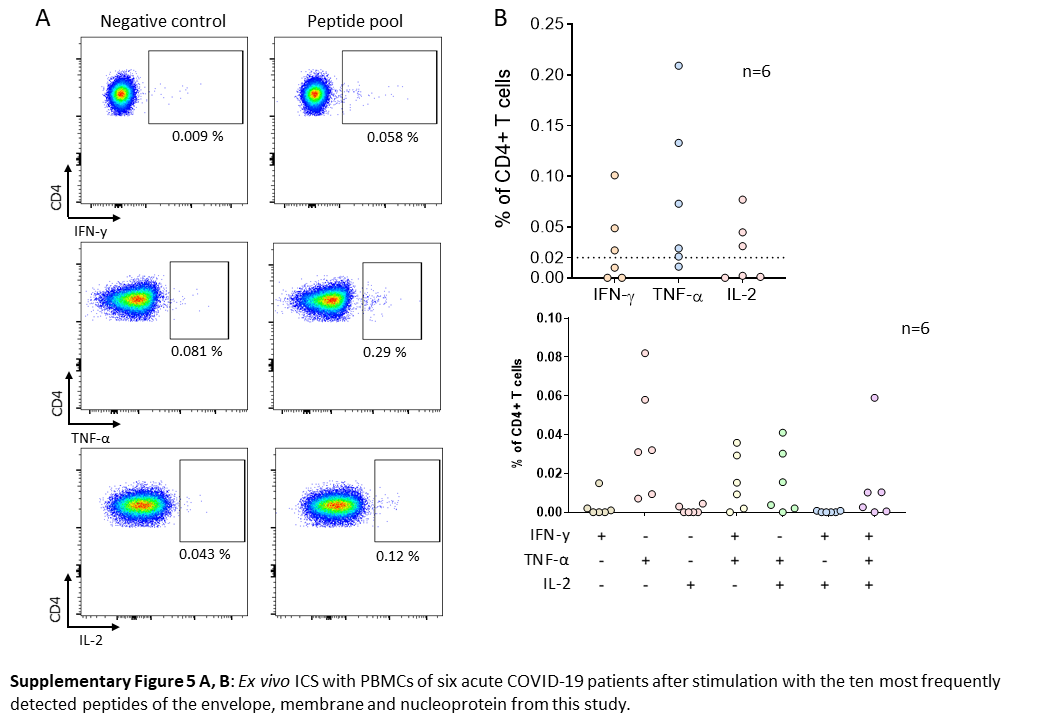

Supplement: S5 Fig — (A) Exemplary IFN-y, TNF-α and IL-2 CD4+ T cell response, pre-gated on CD4+ T cells. (B) Frequencies of IFN-y, TNF-α and IL-2 of CD4+ T cells and the distribution of the frequencies of single, double, and triple positive CD4+ T cells. The threshold for positivity for the cytokines IFN-y, TNF-a and IL-2 was set at 0.02% of all CD4+ T cells. The background detected in the negative controls (R10 + DMSO) was subtracted from positive values. (TIF) [file ppat.1009842.s014.TIF]

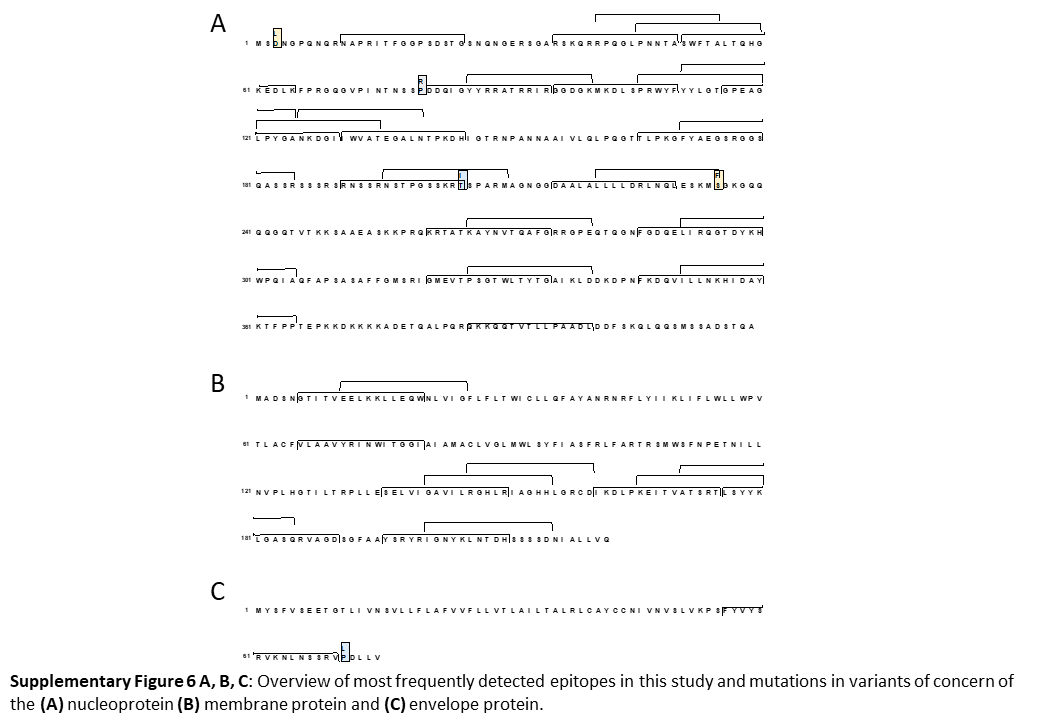

Supplement: S6 Fig — Overview of most frequently detected epitopes in this study and mutations in variants of concern of the (A) nucleoprotein, (B) membrane protein and (C) envelope protein. Horizontal, black parentheses indicate epitopes identified in this study. Red shade indicates asymptomatic CD4+ T cell epitopes identified by Prakash et al. [28] with high conservancy among human and animal coronaviruses. Highlighted amino acids indicate mutations found in variants of concern (according to PANGO lineages: https://cov-lineages.org/). Colour code: yellow, B.1.1.7; light blue, B.1.351; grey, P1 (no VOC-defining mutations are located in the M protein). (TIF) [file ppat.1009842.s015.TIF]
