# Supplementary material for: Broadly directed SARS-CoV-2-specific CD4+ T cell response includes frequently detected peptide specificities within the membrane and nucleoprotein in patients with acute and resolved COVID-19
Source: PLoS Pathog. 2021 Sep 16;17(9):e1009842. doi: 10.1371/journal.ppat.1009842 (PMC8445433; doi:10.1371/journal.ppat.1009842)
Supplement: Attachment — Submitted filename: Point by point-final.docx [file ppat.1009842.s016.docx]

**Part I - Summary**

Please use this section to discuss strengths/weaknesses of study, novelty/significance, general execution and scholarship.

**Reviewer #1:** In the manuscript entitled “Broadly directed SARS-CoV-2-specific CD4+ T cell response includes frequently detected peptide specificities within the membrane and nucleoprotein in patients with acute and resolved COVID-19” by Heide et al., the authors analyze T cell responses (mostly CD4, but also some information on CD8) against the envelope (E), membrane (M) and nucleoprotein (N) in a cohort of 34 patients from Hamburg by performing in vitro stimulation with overlapping peptides. They detect few responses targeting the E-protein, but several responses targeting the M- and N-proteins. The responses against M- and N-derived peptides are almost evenly spread across the proteins and several peptides are targeted by almost all patients in this cohort which lets the authors to conclude that these epitopes can be presented by a number of different HLA-alleles.

Collectively, the authors present an interesting piece of work that certainly adds to the literature regarding SARS-CoV-2-specific immunity. While the study is well performed and the paper is nicely written, I have several comments the authors might want to consider to improve the overall strength of their manuscript.

**We thank reviewer #1 for the benevolent reception and correct interpretation and their justified and insightful suggestions. Please find our detailed point-by-point responses below.**

**Reviewer #2:** In this study by Heide et al, they characterized the breadth and specificity of the SARS-CoV-2 peptide specific response to structural proteins E, M and N in a cohort of 10 acute and 24 convalescent volunteers. They use tried and trusted techniques like the short-term cultured ELISpot to show T cell immunodominant responses at the population (response frequency) and individual (mapped responses) levels. They go further to describe the HLA allotypes restricting these responses and then finally phenotype these responses using ICS. Here, they encounter a challenge as most studies have found in that the detection of SARS-CoV-2 specific responses using ICS is challenging due to the low sensitivity of ICS and the comparatively lower frequencies of circulating SARS-CoV-2 cells in the periphery compared to virus-specific circulating frequencies observed in chronic infections. Their epitope mapping in a group of healthy controls will also help to define source of the cross-reactive epitopes reported in some of the literature.

In summary, their results contribute to the growing body of work identifying SARS-CoV-2 epitopes that could prove to be important addressing cross-reactive epitopes from other infections and in the design of the next generation SARS-CoV-2 vaccines.

**We thank reviewer #2 for the friendly evaluation of the manuscript. We agree, the *ex vivo* magnitude of the T cell response against SARS-CoV-2-specific single peptide specificities is rather low and requires very sensitive methods such as ELISPOT (see Supplementary Figure 1). We discussed the option of applying the AIM assays in the revised manuscript that we are using for other COVID-19 related projects. However, for epitope discovery, we indeed think that antigen-specific *in vitro* expansion followed by the ELISPOT assay, confirmed by ICS is labor some but very sensitive and exact. Of note, the magnitude of the T cell responses detected in healthy controls was much lower (we also added this piece of information to the revised manuscript).**

**Part II – Major Issues: Key Experiments Required for Acceptance**

**Reviewer #1**: Specific points:

The manuscript lacks information on common cold coronaviruses (CCC) and the potential of pre-existing immunity against the analyzed proteins from these CCCs. Could the authors provide information on how closely related the fine-mapped and most targeted epitopes are to the corresponding regions in CCCs?

**Thank you for the constructive and detailed suggestions regarding cross-immunity between SARS-CoV-2 and CCC. In a first step, we performed comprehensive research and investigated the sequence homology between our most frequently targeted epitopes in the E, M, and N protein and corresponding CCC sequences (Mateus et al. 2020; doi: 10.1126/science.abd3871). We agree that this is additional useful information for the community and added a table to the revised manuscript (Supplementary Table 6). Overall, the sequence homology of the majority of frequently detected epitopes was relatively low to intermediate at most. The highest degree of homology was found for Ncl_P22 [range 60-87%].**

**To provide more substantial information on past exposure of our healthy cohort and patients to other CCC, we started to measure IgG antibody responses against the CCC 229E, HKU1, OC43, and NL63 and SARS-CoV-2 in the thawed plasma from 30 patients in our cohort as well as four healthy controls using a commercial recomLine SARS-CoV-2 IgG Immunoblot assay. Indeed, 20 out of 34 individuals showed antibody responses to the nucleoprotein of at least one of four CCC. 24 out of 30 patients showed antibody responses against the nucleoprotein, the receptor-binding domain, or subunit 1 of the spike protein of SARS-CoV-2. Interestingly, the prevalence of IgG antibodies against CCC or SARS-CoV-2 did not correlate with the overall breadth of antigen-specific CD4+ T cell responses (Revision Figure 1).**

**In sum, we provide much more adjunct data on past exposure of the majority of patients to CCC. Even though the general degree of sequence similarity between SARS-CoV-2 specific epitopes seems to be overall low, some degree of cross-reactivity seems to be possible as has been experimentally described for individual epitopes in other studies (Antunes et al., 2021; doi.org/10.1093/infdis/jiab176); Schulien et al., 2021; https://doi.org/10.1038/s41591-020-01143-2). However, for the large number of epitopes detected in this study cross-reacting T cell responses directed against CCC are most unlikely the reason for the vast majority of SARS-CoV-2 specific CD4+ T cell responses. We discuss the new results and cross-reactivity in more depth in the revised manuscript. See also response to reviewer #2 below.**

**Revision Figure 1** Seroprevalence of IgG antibodies against SARS-CoV-2 and CCC. Immunosuppressed patients were excluded from analysis.

I am also unsure about the precise message the authors are trying to convey with regards to HLA restriction. This pertains mostly to the HLA-binding information the authors provide. Some peptides are targeted by almost all patients, irrespective of the patients’ class II HLA alleles. In addition, some patients target several consecutive peptides beyond the mere overlapping regions between two peptides. The authors also discuss these highly promiscuous HLA class II binders, however, the HLA fine-mapping experiments are rather limited in this context. Are these peptides indeed presented by so many HLA molecules?

**We agree. The main finding of our article is the great breadth of the CD4+ T cell response directed against different peptides located within the SARS-CoV-2 E, M, or N protein and we formally determined the HLA restriction experimentally for only three of the epitopes. However, the *in vitro* binding data and the response pattern suggest that a number of peptide epitopes might be restricted by more than one HLA molecule. We toned down the notion of highly promiscuous binding epitopes in the revised manuscript.**

Are the data shown in figure 1B derived ex vivo or after stimulation?

**We apologize for this ambiguity. Figure 3B shows the comparison of the total number of N, M, and E-specific CD4+ T cell responses of each patient after a 10-day *in vitro* cultivation. We have clarified this in the figure legend of Figure 3B of the revised manuscript.**

Were responses in patients (especially resolved patients) stratified by the time post infection? It would be important to see whether there is a correlation. Also, all acute patients were displayed together despite differences in clinical courses (22 mild and 12 severe including 7 with ICU treatment). Did this affect T cell responses? Were they sampled early? Have they already received ICU treatment/steroids?

**We have further analyzed and listed the detailed clinical status, specific therapy, and time after infection of all patients in correlation to the number of CD4+ T cell epitopes detected. We did not find a correlation between the time after infection and the number (nor magnitude) of the CD4+ T cell response (Revision Figure 2). We also compared the disease severity and the CD4+ T cell response and did not find a significant difference (Revision Figure 2). However, this current paper was mainly devised for epitope discovery. We suspect that there are some correlations between the parameters mentioned by reviewer #1 – but larger cohorts and multivariate analysis would be needed to tease out any correlation. We added this limitation of the current study to the discussion of the revised manuscript. We also added the specific COVID-19 therapy status (Remdesivir, Tocilizumab, steroids) to our revised Supplementary Table 1. However, we do not think that the priming and detection of T cell responses were affected greatly by any of the COVID-19 therapy regimens. Indeed, we detected T cell responses in all immune-suppressed patients.**

In Figure 3B, it would be helpful to see the original FACS plots from the HLA-restriction experiments.

**Revision Figure 2 A:** No correlation between the number of CD4+ T cell responses and time post infection. **B)** No significant influence of disease severity nor immunosuppressive treatment (COVID-19-specific treatment and chemotherapy) on the number of CD4+ T cell responses.

**We agree. We have added all of the original FACS plots from the HLA-restriction experiments to Supplementary Figure 3. We also developed an HLA-II tetramer for Mem_aa145-164 (LRGHLRIAGHHLGRCDIKDL) (HLA-DRB*11) which confirms the experimentally determined HLA restriction (revised Figure 6).**

The authors discuss that “we detected no significant difference between the number and magnitude of T cell responses in early acute vs. resolved infection” (lines 267f), however, in table 2, it appears as if aCov patients had fewer responses against membrane compared to rCov patients (at least in the responses between peptides 5 and 25). Can this be quantified?

**For comparison between the number of CD4+ T cell responses in acute and resolved patients, we conducted a student’s t-test (Revision Figure 3). No significant differences were found. We added this information to the revised manuscript (Figure 3B). Also, see response above – we suspect that large cohorts and more sophisticated analysis to rule out potential confounders would be needed to tease out any such trend.**

**Revision Figure 3** Comparison between the number of CD4+ T cell responses in COVID-19 patients with acute and resolved infection.

Why were the additional patients recruited “for investigation of ex vivo responses” (line 341) not listed in table 1? Were the uninfected healthy controls historic controls before the beginning of the pandemic?

**Indeed, we now list the detailed patient information of the six patients studied only for their *ex vivo* response in Supplementary Table 1. Originally, we only wanted to list data of the patients for whom we comprehensively determined the breadth of the SARS-CoV-2 M, N, and E peptide response.**

**All healthy control samples were collected either before, or at the very early stages of the pandemic (March 2020 – May 2020). All healthy controls had no history of symptoms or contact with infected individuals and were seronegative. Of note, the prevalence during this time in Hamburg was lower than 1 % (Brehm et al., 2021; https://doi.org/10.1038/s41598-021-85081-0). The newly generated serological immunoblot data measuring serological response against CCC and SARS-CoV-2 for four healthy participants demonstrate previous exposure to CCC but no IgG against SARS-CoV-2 in any of the subjects**.

I am not convinced that we are truly looking at a Th1 bias in these cells, the provided data are too limited in my view to draw this conclusion.

**We agree that our results do not exclude the possibility of a Th2 response. We toned down any notion of a Th1 bias in the revised manuscript and changed the according chapter.**

The variants of concern should be classified following the new WHO label.

**We updated the nomenclature of the variants of concern and added the delta variant to our discussion.**

**Reviewer #2:** (No Response)

**Part III – Minor Issues: Editorial and Data Presentation Modifications**

**Reviewer #1:** (No Response)

**Reviewer #2:** 1. The manuscript was easy to read and well-written however, the figures could be improved with more granularity in terms of information e.g. immunosuppressed patients and acute vs convalescent subject in the cohort could be distinguished using different colours or shape for in-depth information in figure 1B.

**Great idea! We added your suggestion to Figure 3B and also improved the layout of all figures and tables.**

2. Line 55 – needs an update

**We updated the numbers of cases and death due to COVID-19 and will make sure to update it again before publication – Thank you.**

3. Line 57 – can authors explain what they mean by ‘general fragility’ as a risk factor for severe disease and why co-morbidities does not suffice

**We changed the sentence to “with the main established risk factors being old age and comorbidities”.**

4. Consider using convalescence instead of reconvalescence.

**We changed it to covalescence throughout the whole manuscript.**

5. Table 1 – what do the range under blood count, immunolog,y and clinical parameters at the time of analysis represent? Are these the range in healthy controls or range in their cohort. Also, why is there none of this information for the resolved COVID-19 cohort?

**The values are indeed the range in healthy controls – we added this information to the revised Table 1. We have to explain: In all tables, we only show laboratory values that were taken at the same time as the blood samples for our study. Since many convalescent patients were already healthy, no laboratory values were determined. Also, see the revised manuscript.**

6. Consider changing label for Y-axis on Figure 1B for clarity. e.g. number of CD4+ responses in each subject.

**We changed the label for the Y-axis accordingly. We thank reviewer #2 for this suggestion.**

7. Why were 4 patients excluded in Figure 1B?

**For the N-protein, only 30 patients out of 34 were tested. This is also shown in Figure 1 of the revised manuscript. When we started our investigation with the first four patients (aCov-1, rCov 1-3), the nucleoprotein-specific peptides had not arrived, yet. For the E and M protein, all 34 patient samples were examined for the CD4+ T cell response, and no patient was excluded for Figure 3B. We apologize for causing any confusion and added this information to the figure legend of Figure 4.**

8. Add a reference to supplementary table 1 in text for summary data in lines 157-160

**The reference has been added to the revised manuscript.**

9. Figure 1A and Supplementary figure 2 and 3A - What are cytokine gates set based on? Unstimulated (negative) controls or FMO? If negative controls, did they use the R1O or DMSO control? DMSO control would be the preferred control for setting gates here. This information should be clear in the figure legends. Gating in supplementary figure 2 seems to be quite stringent to pick up strong responders, this makes for robust data however the rationale for this should be clearly explained in text. Responses like NCL-P11 amongst others appear low to be a ‘true positive’. How did the authors confirm these extremely low responses? Concerns would be around this inflating the response frequency reported in the manuscript.

**All cytokine gates are set based on negative controls that were treated with R10 + DMSO. We added this information to the respective figure legends.**

**We agree that intracellular cytokine staining by flow cytometry is not the most sensitive method for the ex vivo detection of antigen-specific T cells. We therefore tested all single peptides with ELISPOT, one of the most sensitive methods. Only if we saw a distinct response in the ELISPOT assay (three times as many spots as the negative control (R10+DMSO) and at least a total number of 30 spots /30.000 cells), we tested the IFN-y response after stimulation with the respective single peptide in the ICS assay. If this response in the ICS assay was above 0.02% of all CD4+ T cells and three times as big as the negative control, it was counted as a positive peptide response. For clarification: in each ICS assay, we deconvoluted positive peptide pools and several COVID-19 peptides tested negative and several positive controls after stimulation with PMA and ionomycin were also run. This approach is well-established (Schulze zur Wiesch et al., 2005; https://doi.org/10.4049/jimmunol.175.6.3603, Day et al., 2003; DOI: 10.1172/JCI18509)**

10. Supplementary figure 3A – what did the full T cell flow cytometer panel look like. A supplementary table will be panel will be useful. Authors report that the responses are Th1 however, it seems the panel was set up to only detect Th1-biased responses (Ifng, Tnf, IL-2) in the first place. Were other cytokines used and not detected? Perhaps clarify that they had sought to describe Th1 responses as the assay was not set up to detect any other Th-biased response other than Th1. While I acknowledge that non-Th1 cytokines can be difficult to assay ex vivo with standard ICS and may have informed the panel design and result presentation, author’s wordings suggest that other Th subsets were evaluated and not found. If other Th phenotypes are desired, then a cytokine independent approach e.g., AIM assay would be the way to go. The AIM assay is also more sensitive than the ICS and would be useful in quantifying the magnitude of these responses.

**We agree with reviewer #2. Our ICS panel which is now shown in Supplementary Table 9 was biased towards the detection of a Th1 cytokine pattern. We are well-aware of the possibility of other T cell cytokine signatures (Zhao et al., 2021; https://pubmed.ncbi.nlm.nih.gov/ 33622974/).**

**We added the information to the revised manuscript that we did not look at Th2 or other cytokine signatures. We also discussed the use of the AIM assay during the revision process which we set up for other projects, but we indeed think that for epitope mapping - which was the main goal of this article - *in vitro* expansion using Il-2 and IFN-y ICS are a very precise and well-established approach. Therefore, this article is based on intracellular cytokine staining. However, the peptide-specificities detected in this study will be useful for future *ex vivo* investigations with the AIM assay.**

11. Flow cytometry - threshold for positivity for Ifng+, Tnf+, and IL-2+ should be mentioned in main text.

**The threshold for positivity for the cytokines IFN-y, TNF-a and IL-2 was set at 0.02% of all CD4+ T cells. The background detected in the negative controls (R10 + DMSO) was subtracted from positive values. We added this information to the material and methods section as well as to the main text of the revised manuscript.**

12. Line 268 which addresses evolution of response at a population level could be made into a summary table

**We did not find a clear or statistically significant correlation between the time after infection and the number (nor magnitude) of the CD4+ T cell response (Revision Figure 2A). We also compared the disease severity and the CD4+ T cell response and did not find a significant difference (Revision Figure 2B). We suspect that there are correlations between parameters such as the timepoint after infection as well as the disease severity but our cohort of 34 patients is too small and heterogenous for this kind of analysis – larger cohorts and multivariate analysis would be needed to prove these correlations. We added this limitation of the current study to the revised manuscript.**

13. Line 282 – update to new WHO names for variants of concern (VOC)

**We updated the nomenclature of all VOC.**

14. Are there any experiments evaluating the change, if any, of the T cell responses when peptides covering mutated regions of VOCs are used to re-stimulate short term cell lines?

**The mutated regions lie nearly exclusively outside the described epitopes here (Supplementary Figure 5). In other words, it is unlikely that T cell escape plays a major role as has been previously described (Geers et al, 2021; DOI: 10.1126/sciimmunol.abj1750). We therefore did not focus our experiments on variants but admit that this would be another interesting aspect that we now discuss in the revised manuscript.**

15. Line 315 – 317 – the authors need to be careful about this statement. I do not believe their data has the power to confirm this so it is speculative and may be stretching their conclusions.

**We toned this statement down in the revised manuscript, but we do see that B-cell depleted patients show a broadly directed SARS-CoV-2 T cell response (Malsy et al., https://academic.oup.com/cid/advance-article/doi/10.1093/cid/ciaa1637/5939946). This was part of the original discussion.**

16. Typo in table 1 – Days sinc(y)e start of symptoms

**We corrected this typo, thank you!**

17. Supplementary table 1 - Consider ordering this based on clinical phenotypes. Perhaps add nationality to the table

**We will upload this table as Excel sheet with filter function, this will allow to change the order of all patient data by the individual user. We also added the respective nationality of each patient to the table.**

18. Did country of origin play a role in study findings e.g. on the breadth of responses and do T cell responses targeting particular epitopes impact on recovery or associate with VL?

**The nationality of the patients did not seem to have influenced the breadth or specificity of the SARS-CoV-2-specific CD4+ T cell response in our cohort (Revision Figure 4), but it has to be kept in mind that our study cohort is too small and heterogenous for this kind of analysis. We also did not find particular SARS-CoV-2 peptide responses to be correlated with any other parameter like the clinical course. Again, the main focus of this current study was the discovery of SARS-CoV-2 CD4+ T cell epitopes, unfortunately our cohort is too small for some of these analyses.**

**Revision Figure 4** No difference between the number of CD4+ T cell response and the origin in our study cohort (n=34).

19. Did authors check for antibody or T cell responses to CCC in this cohort?

**Indeed. For the revised manuscript, we measured IgG antibody responses to the nucleoprotein of the four CCC NP_229E, NP_HKU1, NP_OC43, and NP_NL63 using a commercial recomLine SARS-CoV-2 IgG Immunoblot assay to the nucleoprotein, receptor binding domain and the S1 subunit of the spike protein. This data demonstrates the prevalence of an antibody-response against CCC that might be linked to cross-immunity against SARS-CoV-2. We added this information to the revised manuscript as Supplementary Table 7.**

20. Line 321 – Can the authors comment on the incidence of re-infection after natural infection vs after vaccination given the breadth of the responses following natural infection?

**See response above and revised discussion. The broad SARS-CoV-2 M, N, and E-specific CD4+ T cell memory response generated during a first course should shorten and alleviate a possible re-infection. The vaccine induced Ab response alone seems to render (near) sterile immunity.**

21. The healthy control data seems to stand alone. Can they integrate this into their data e.g were there shared epitopes? Are shared responses targeting conserved regions? Were they expanded by infection?

**We found very few responses in our healthy control group that were overall of low magnitude (Revision Figure 5 B). All peptide responses that we found in our healthy control cohort were also shared in the patient cohort (Revision Figure 5A). Since very few mutations have been described within the E, M and N protein, all these peptide responses are located in conserved regions. Revision Figure 5B shows the highest percentage of IFN-y+ CD4+ T cells for each patient. The overall magnitude of the CD4+ T cell response was significantly smaller in our healthy control group compared to the acute and resolved patients demonstrating that the T cell response is expanded by SARS-CoV-2 infection. We discuss this in the revised manuscript.**

**Revision Figure 5 A** Response frequency of the patient cohort (n=30) and the healthy cohort (n=12). **B** Comparison of the highest IFN-y+CD4+ response from each patient. The magnitude of the healthy control group was significantly lower.

PLOS authors have the option to publish the peer review history of their article (what does this mean?). If published, this will include your full peer review and any attached files.

**We are very glad about the constructive suggestions from both reviewers and would like to publish the peer review history which helped improve our manuscript.**

If you choose “no”, your identity will remain anonymous but your review may still be made public.

Do you want your identity to be public for this peer review? For information about this choice, including consent withdrawal, please see our Privacy Policy.

Reviewer #1: No

Reviewer #2: No
